# RETRACTED: Yang et al. Design and Dynamic Simulation of a Novel Traveling Wave Linear Ultrasonic Motor. *Micromachines* 2022, *13*, 557

**DOI:** 10.3390/mi16091041

**Published:** 2025-09-11

**Authors:** Lin Yang, Kaixi Yao, Weihao Ren, Liang Chen, Mojian Yang, Rongcheng Zhao, Siyu Tang

**Affiliations:** 1 State Key Laboratory of Mechanics and Control of Mechanical Structures, Nanjing University of Aeronautics and Astronautics, Nanjing 211106, China; yanglin@nuaa.edu.cn (L.Y.); cathyyao@nuaa.edu.cn (K.Y.); 2 Innovation Academy for Microsatellites of Chinese Academy of Sciences, Shanghai 201210, China; chen15951991306@163.com; 3 Nuaa Super Control Technology Co., Ltd., Nanjing 211100, China; yangmojian@126.com (M.Y.); rch_zhao@126.com (R.Z.); tangsiyu1217@126.com (S.T.)

The journal retracts the article “Design and Dynamic Simulation of a Novel Traveling Wave Linear Ultrasonic Motor” [1].

Following the article’s publication, the authors contacted the editorial office regarding the concerns related to biased simulation settings and the accuracy of the data presented in this study [1].

Adhering to our standard procedure, an investigation was conducted by the Editorial Office and the Editorial Board that confirmed the presence of a scientific error that created a significant mismatch between the simulation and experimental outcomes, particularly affecting the friction and contact behavior between stator and mover. As a result, the Editorial Board and the authors agreed that these errors undermine the validity of the overall findings presented in the article, and therefore have decided to retract this article as per MDPI’s retraction policy (https://www.mdpi.com/ethics#_bookmark30).

This retraction was approved by the Section Editor-in-Chief *Micromachines*.

The authors agreed to this retraction.
